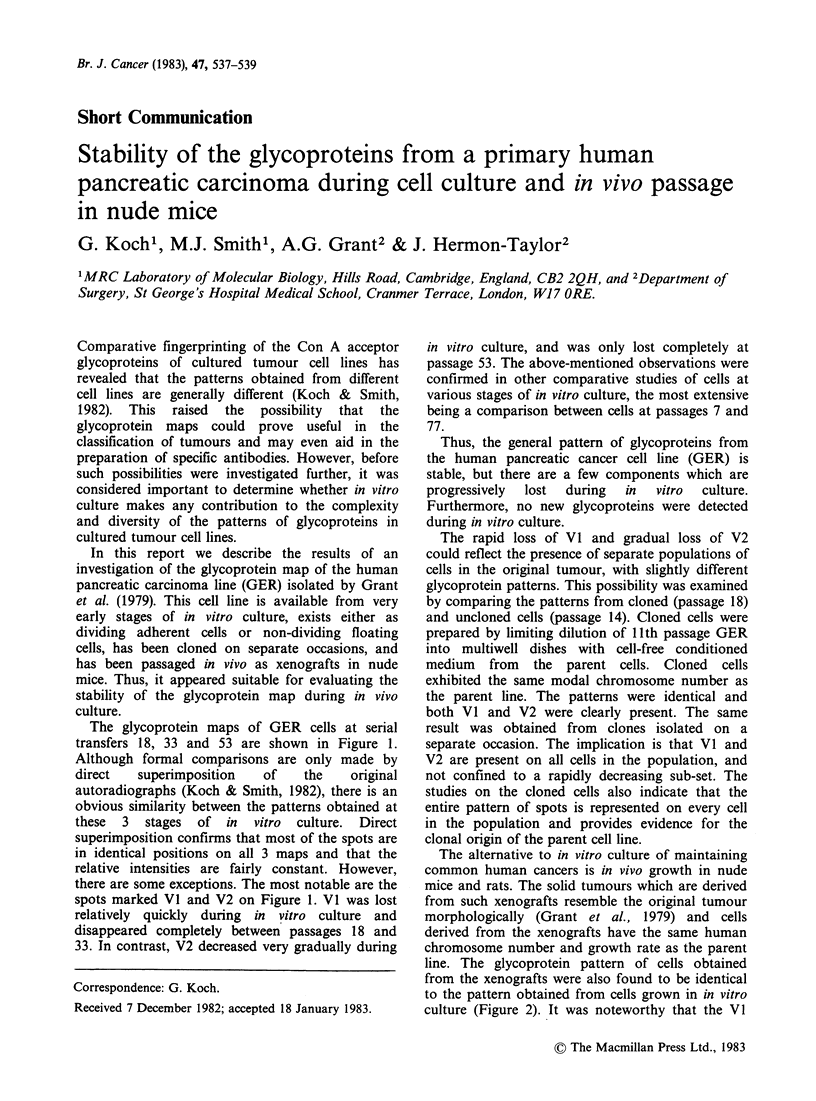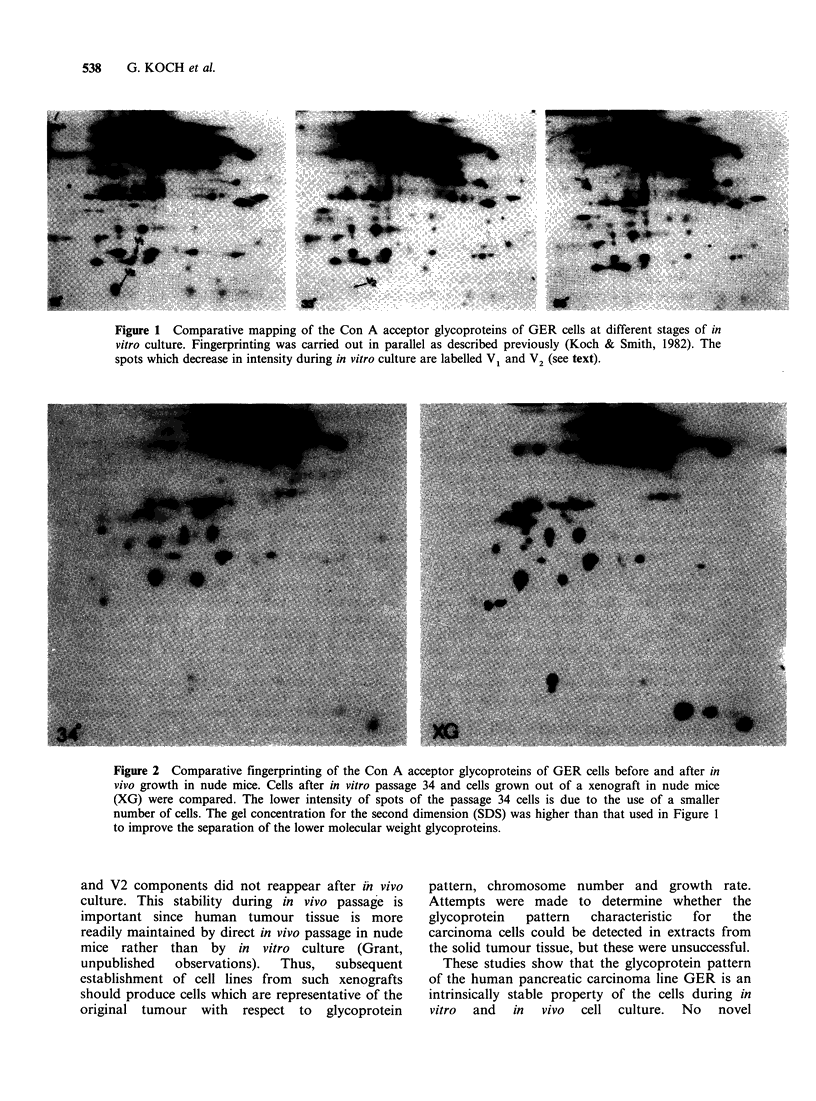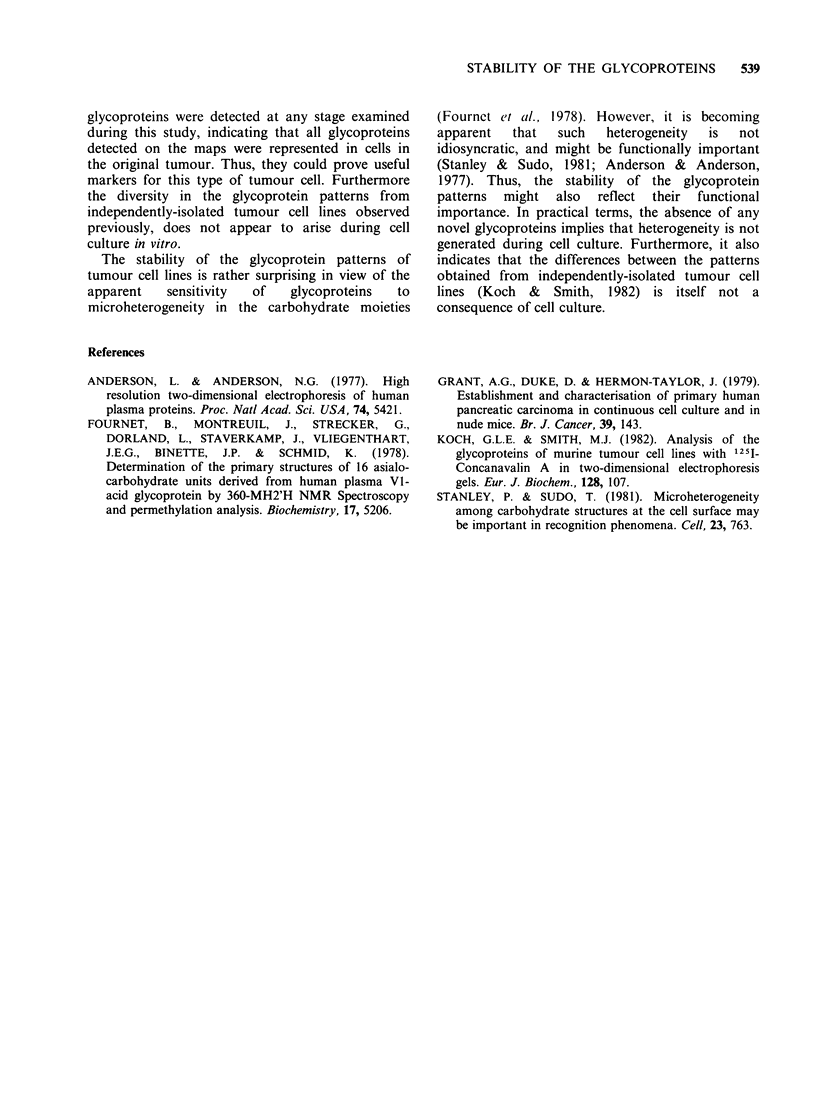# Stability of the glycoproteins from a primary human pancreatic carcinoma during cell culture and in vivo passage in nude mice.

**DOI:** 10.1038/bjc.1983.84

**Published:** 1983-04

**Authors:** G. Koch, M. J. Smith, A. G. Grant, J. Hermon-Taylor

## Abstract

**Images:**


Br. J. Cancer (1983), 47, 537–539

**Short Communication**

### Stability of the glycoproteins from a primary human pancreatic carcinoma during cell culture and *in vivo* passage in nude mice

G. Koch[1], M.J. Smith[1], A.G. Grant[2] & J. Hermon-Taylor[2]

[1]*MRC Laboratory of Molecular Biology, Hills Road, Cambridge, England, CB2 2QH, and* [2]*Department of Surgery, St George's Hospital Medical School, Cranmer Terrace, London, W17 0RE.*

Comparative fingerprinting of the Con A acceptor glycoproteins of cultured tumour cell lines has revealed that the patterns obtained from different cell lines are generally different (Koch & Smith, 1982). This raised the possibility that the glycoprotein maps could prove useful in the classification of tumours and may even aid in the preparation of specific antibodies. However, before such possibilities were investigated further, it was considered important to determine whether *in vitro* culture makes any contribution to the complexity and diversity of the patterns of glycoproteins in cultured tumour cell lines.

In this report we describe the results of an investigation of the glycoprotein map of the human pancreatic carcinoma line (GER) isolated by Grant *et al.* (1979). This cell line is available from very early stages of *in vitro* culture, exists either as dividing adherent cells or non-dividing floating cells, has been cloned on separate occasions, and has been passaged *in vivo* as xenografts in nude mice. Thus, it appeared suitable for evaluating the stability of the glycoprotein map during *in vivo* culture.

The glycoprotein maps of GER cells at serial transfers 18, 33 and 53 are shown in Figure 1. Although formal comparisons are only made by direct superimposition of the original autoradiographs (Koch & Smith, 1982), there is an obvious similarity between the patterns obtained at these 3 stages of *in vitro* culture. Direct superimposition confirms that most of the spots are in identical positions on all 3 maps and that the relative intensities are fairly constant. However, there are some exceptions. The most notable are the spots marked V1 and V2 on Figure 1. V1 was lost relatively quickly during *in vitro* culture and disappeared completely between passages 18 and 33. In contrast, V2 decreased very gradually during *in vitro* culture, and was only lost completely at passage 53. The above-mentioned observations were confirmed in other comparative studies of cells at various stages of *in vitro* culture, the most extensive being a comparison between cells at passages 7 and 77.

Thus, the general pattern of glycoproteins from the human pancreatic cancer cell line (GER) is stable, but there are a few components which are progressively lost during *in vitro* culture. Furthermore, no new glycoproteins were detected during *in vitro* culture.

The rapid loss of V1 and gradual loss of V2 could reflect the presence of separate populations of cells in the original tumour, with slightly different glycoprotein patterns. This possibility was examined by comparing the patterns from cloned (passage 18) and uncloned cells (passage 14). Cloned cells were prepared by limiting dilution of 11th passage GER into multiwell dishes with cell-free conditioned medium from the parent cells. Cloned cells exhibited the same modal chromosome number as the parent line. The patterns were identical and both V1 and V2 were clearly present. The same result was obtained from clones isolated on a separate occasion. The implication is that V1 and V2 are present on all cells in the population, and not confined to a rapidly decreasing sub-set. The studies on the cloned cells also indicate that the entire pattern of spots is represented on every cell in the population and provides evidence for the clonal origin of the parent cell line.

The alternative to *in vitro* culture of maintaining common human cancers is *in vivo* growth in nude mice and rats. The solid tumours which are derived from such xenografts resemble the original tumour morphologically (Grant *et al.,* 1979) and cells derived from the xenografts have the same human chromosome number and growth rate as the parent line. The glycoprotein pattern of cells obtained from the xenografts were also found to be identical to the pattern obtained from cells grown in *in vitro* culture (Figure 2). It was noteworthy that the V1

Correspondence: G. Koch.

Received 7 December 1982; accepted 18 January 1983.

© The Macmillan Press Ltd., 1983

**Figure 1** Comparative mapping of the Con A acceptor glycoproteins of GER cells at different stages of *in vitro* culture. Fingerprinting was carried out in parallel as described previously (Koch & Smith, 1982). The spots which decrease in intensity during *in vitro* culture are labelled $V_1$ and $V_2$ (see **text**).

**Figure 2** Comparative fingerprinting of the Con A acceptor glycoproteins of GER cells before and after *in vivo* growth in nude mice. Cells after *in vitro* passage 34 and cells grown out of a xenograft in nude mice (XG) were compared. The lower intensity of spots of the passage 34 cells is due to the use of a smaller number of cells. The gel concentration for the second dimension (SDS) was higher than that used in Figure 1 to improve the separation of the lower molecular weight glycoproteins.

and V2 components did not reappear after *in vivo* culture. This stability during *in vivo* passage is important since human tumour tissue is more readily maintained by direct *in vivo* passage in nude mice rather than by *in vitro* culture (Grant, unpublished observations). Thus, subsequent establishment of cell lines from such xenografts should produce cells which are representative of the original tumour with respect to glycoprotein pattern, chromosome number and growth rate. Attempts were made to determine whether the glycoprotein pattern characteristic for the carcinoma cells could be detected in extracts from the solid tumour tissue, but these were unsuccessful.

These studies show that the glycoprotein pattern of the human pancreatic carcinoma line GER is an intrinsically stable property of the cells during *in vitro* and *in vivo* cell culture. No novel glycoproteins were detected at any stage examined during this study, indicating that all glycoproteins detected on the maps were represented in cells in the original tumour. Thus, they could prove useful markers for this type of tumour cell. Furthermore the diversity in the glycoprotein patterns from independently-isolated tumour cell lines observed previously, does not appear to arise during cell culture *in vitro*.

The stability of the glycoprotein patterns of tumour cell lines is rather surprising in view of the apparent sensitivity of glycoproteins to microheterogeneity in the carbohydrate moieties (Fournet *et al.*, 1978). However, it is becoming apparent that such heterogeneity is not idiosyncratic, and might be functionally important (Stanley & Sudo, 1981; Anderson & Anderson, 1977). Thus, the stability of the glycoprotein patterns might also reflect their functional importance. In practical terms, the absence of any novel glycoproteins implies that heterogeneity is not generated during cell culture. Furthermore, it also indicates that the differences between the patterns obtained from independently-isolated tumour cell lines (Koch & Smith, 1982) is itself not a consequence of cell culture.

### References

ANDERSON, L. & ANDERSON, N.G. (1977). High resolution two-dimensional electrophoresis of human plasma proteins. *Proc. Natl Acad. Sci. USA*, **74**, 5421.

FOURNET, B., MONTREUIL, J., STRECKER, G., DORLAND, L., STAVERKAMP, J., VLIEGENTHART, J.E.G., BINETTE, J.P. & SCHMID, K. (1978). Determination of the primary structures of 16 asialo-carbohydrate units derived from human plasma V1-acid glycoprotein by 360-MH2'H NMR Spectroscopy and permethylation analysis. *Biochemistry*, **17**, 5206.

GRANT, A.G., DUKE, D. & HERMON-TAYLOR, J. (1979). Establishment and characterisation of primary human pancreatic carcinoma in continuous cell culture and in nude mice. *Br. J. Cancer*, **39**, 143.

KOCH, G.L.E. & SMITH, M.J. (1982). Analysis of the glycoproteins of murine tumour cell lines with $^{125}$I-Concanavalin A in two-dimensional electrophoresis gels. *Eur. J. Biochem.*, **128**, 107.

STANLEY, P. & SUDO, T. (1981). Microheterogeneity among carbohydrate structures at the cell surface may be important in recognition phenomena. *Cell*, **23**, 763.